# The Emergent Power of Human Cellular vs Mouse Models in Translational Hair Research

**DOI:** 10.1093/stcltm/szac059

**Published:** 2022-08-13

**Authors:** Ana Rita Castro, Carlos Portinha, Elsa Logarinho

**Affiliations:** Aging and Aneuploidy Group, IBMC, Instituto de Biologia Molecular e Celular, Universidade do Porto, Porto, Portugal; i3S, Instituto de Investigação e Inovação em Saúde, Universidade do Porto, Porto, Portugal; Saúde Viável—Insparya Hair Center, Porto, Portugal; Doctoral Program in Biomedical Engineering, Faculdade de Engenharia, Universidade do Porto, Porto, Portugal; Saúde Viável—Insparya Hair Center, Porto, Portugal; Aging and Aneuploidy Group, IBMC, Instituto de Biologia Molecular e Celular, Universidade do Porto, Porto, Portugal; i3S, Instituto de Investigação e Inovação em Saúde, Universidade do Porto, Porto, Portugal; Saúde Viável—Insparya Hair Center, Porto, Portugal

**Keywords:** hair loss, hair follicles, translational research, mouse models, human models

## Abstract

Different animal models have been used for hair research and regeneration studies based on the similarities between animal and human skins. Primary knowledge on hair follicle (HF) biology has arisen from research using mouse models baring spontaneous or genetically engineered mutations. These studies have been crucial for the discovery of genes underlying human hair cycle control and hair loss disorders. Yet, researchers have become increasingly aware that there are distinct architectural and cellular features between the mouse and human HFs, which might limit the translation of findings in the mouse models. Thus, it is enticing to reason that the spotlight on mouse models and the unwillingness to adapt to the human archetype have been hampering the emergence of the long-awaited human hair loss cure. Here, we provide an overview of the major limitations of the mainstream mouse models for human hair loss research, and we underpin a future course of action using human cell bioengineered models and the emergent artificial intelligence.

## Graphical Abstract

**Figure:**
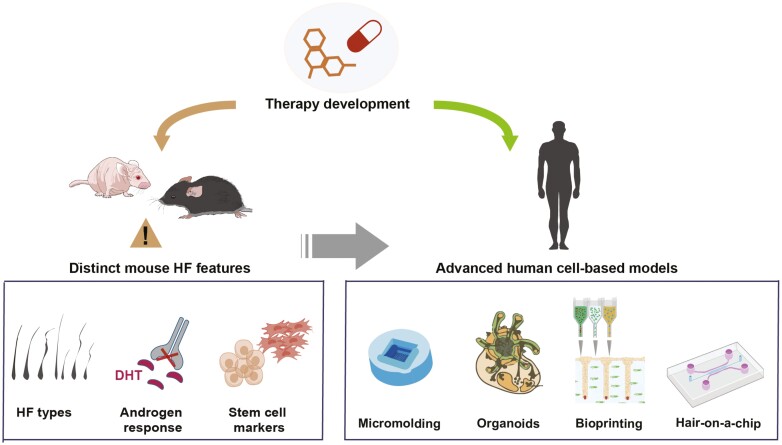

**Significance Statement**

This review aims to comprehensively compile the differences between mouse and human hair biology, and their implications in translational studies for hair loss therapy. We start by appraising several mouse models used in hair research studies and by underlining major distinctive features between human and mouse hair. We then discuss the limitations of the mainstream mouse models and how human cell-based approaches start being prioritized for high-throughput drug screenings and bioengineering solutions toward the development of effective therapies against alopecia.

## Introduction

Human hair follicle (HF) function and regulation are not yet completely understood. The main reasons behind this are: the inability to manipulate human HFs in vivo and the HFs’ scarcity for in vitro and ex vivo studies. The major advancements in the HF research field have primarily arisen from the use of in vitro and in vivo animal models, including rats, hamsters, rabbits, sheep, monkeys, and mainly mice. Natural or genetically engineered loss-of-function and gain-of-function mice have been extensively used to understand the molecular mechanisms controlling HF morphogenesis and cyclic growth, which provided an invaluable contribution to the understanding of hair loss disorders. Moreover, mice models have been crucial to test the efficiency and safety of pharmacological and bioengineering treatments. Yet, one major caveat has been the differences between animal and human hair, which may explain why human hair loss disorders are still seeking for effective therapeutic strategies. Whereas stem cell-based therapy has proven relatively effective in mouse models, it remains intangible to humans. The probable reason behind this is the distinctive biological identity of the human HF that makes it an incredibly unique organ. Future research should extensively characterize the human HF cell populations and their microenvironment to come up with regenerative therapies able to tackle hair loss disorders.^1^

## Mouse Models for Hair Research

Different animal models have been used for hair loss and regrowth studies in vivo, including mice (reviewed in Porter^2^), rats,^3^ goats,^4^ and monkeys.^5^ Despite being more divergent from humans than other models, mice have been predominantly used in hair research due to their easier handling, well-established protocols and ethical approvals. Several mouse strains are currently used for hair growth studies,^6^ the most popular being *C57BL/6* and *C3H*, whose pelage (but not ear and tail) skin pigmentation is merely dependent on their follicular melanocytes.^7^ This way, anagen cycles of hair growth are easily detected by the darkening of shaved skin.^8^ Up to date, foremost knowledge on HF biology and cycling has arisen from studies using spontaneous and/or genetically engineered mouse mutants with hair loss outcome.^9^ Some relevant examples of spontaneous mutants exhibiting HF defects are listed in Table 1. These spontaneous mouse mutants significantly contributed to the identification of new genes involved in hair loss, as well as triggered further research on transgenic mouse models.^2^ Accordingly, a continuously growing number of genetically engineered mutants unveiled the mechanisms controlling hair morphogenesis, cycle, and pigmentation (reviewed in Nakamura et al^10^). Although these models provided valuable knowledge to conceive hair loss therapeutic interventions, inconsistent outcomes have often arisen when conveyed into a human background. Attention should be given to species-specific differences.

**Table 1.** Mouse models with hair loss.

Mutant strain	Mutant gene/origin	Phenotype	Reference
*ragged/opossum* (Raop)	Mutation in the Sox18 transcription factor gene	Reduced number of follicles	^11^
*waved 2* (*Egfrwa2*)	Mutation in the EGF receptor tyrosine kinase	Abnormal hair morphogenesis	^12^
*hairless* (*Hr hr*)	Hypomorphic mutation in the hairless gene	Severe abnormalities during the first catagen and total alopecia	^13^
*Nude* (*Foxn1*^*nu*^)	Nude allele Foxn1^nu^ in Foxn1 transcription factor gene	Complete hair loss	^14^
*balding* (*Dsg3*^*bal*^)	Mutation in the Desmoglein-3 gene	Abnormal hair shaft structure	^15^
B6CBAF1	Hybrid cross between C57BL/6 female and CBA male	Testosterone inducible model of alopecia	^16^

## Mouse vs Human Hair

**Figure 1.**
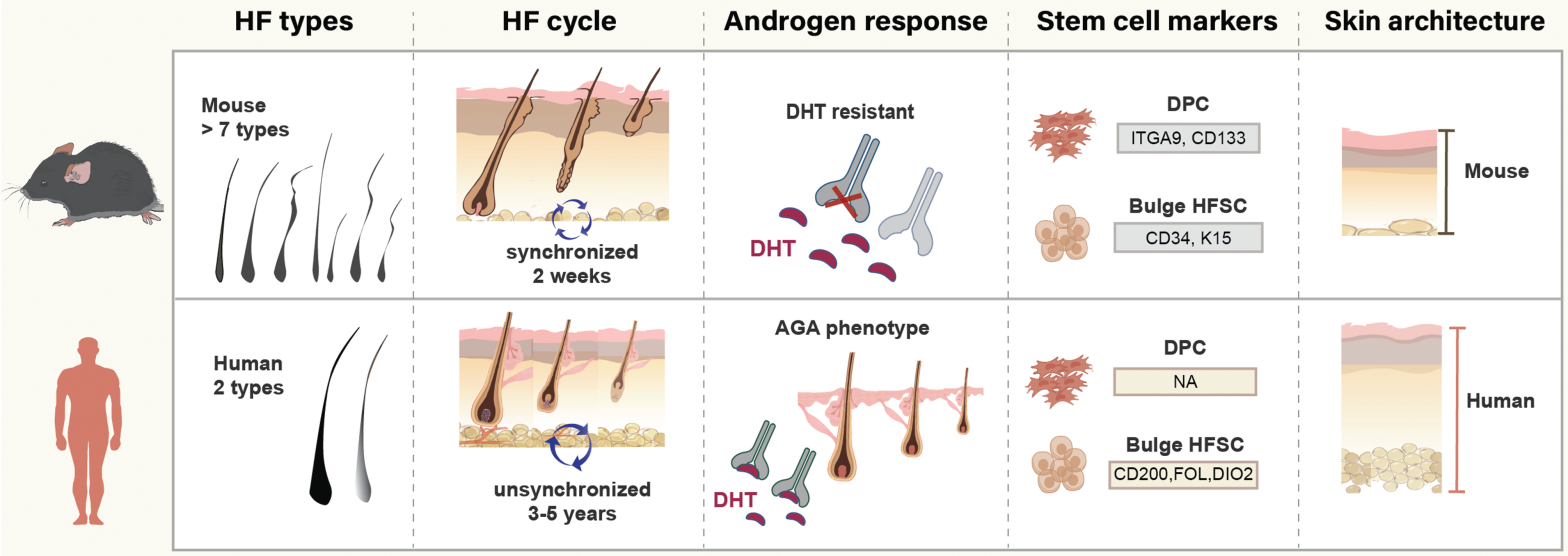
Schematic summary of the main differences between mouse and human HFs. Mice have many different types of HFs whereas humans only have two (terminal pigmented and vellus non-pigmented). Anagen growth phase takes 2 weeks in mice compared to 3-5 years in humans. DP moves upwards to contact the stem cell niche during mice catagen, which is not observed during the human hair cycle. Mice do not exhibit AGA, which in humans is due to the expression of androgen receptors in the DP. The DP and bulge stem cell niches’ biomarkers are distinct between mice and humans. Despite their similar structural layering, human and mouse skins differ in thickness and stiffness. Abbreviations: AGA, androgenetic alopecia; DP, dermal papilla; HFs, hair follicles.

Major differences exist between mouse and human hair, namely in the HF morphogenesis, cycling, structure, and microenvironment (eg, immune and hormonal regulation) (Fig. 1). First, HF function in humans is vastly different from its role in other mammals. Whereas the fur primarily controls thermoregulation in animals, the human HFs mainly protect the scalp skin from ultraviolet radiation rather than acting as heat isolators.^17^ Second, hair cycle behavior and growth are noticeable different in mice and humans: (i) anagen growth phase takes 2 weeks in mice compared to 3-5 years in human^18,19^; (ii) hair shaft shedding (exogen) is a well-controlled process in mice, in which old hair shafts are kept for several HF cycles^20^; (iii) contrarily to rodents, human hair cycle occurs asynchronously in the scalp.^18,21^ In addition, the hair types are also different between humans and mice. Adult humans have two major hair types visible on the scalp, terminal hairs (pigmented) and vellus hairs (thin, non-pigmented).^22^ Melanin is transferred to the hair fiber cortex in humans, whereas primarily to the hair fiber medulla in mice.^7^ Mice have many different hair types, namely truncal (pelage) hairs, vibrissae, muzzle hairs, tail, cilia, and perianal hair.^23^ These hair types should be deeply investigated in hair studies since distinct molecular mechanisms orchestrate different HF populations.^24^ Besides, the mouse hair does not transit from a vellus to terminal hair. Regarding the hair structure during cycling, mouse and human do not differ substantially, with human scalp follicles showing larger and longer hair shafts than mouse pelage follicles. However, in vibrissae follicles the dermal papilla (DP) never reaches the telogenic bulge, making it hard to explain hair follicle stem cell (HFSC) migration from the bulge to the follicular base during anagen onset.^25^ HFSCs are a pool of quiescent multipotent stem cells in the outermost layer of the HF which are activated by the DP to proliferate and promote hair growth.

Moreover, androgenic effects in mice HF proved quite distinct from that observed in human scalp HF. The most common form of human hair loss is androgenetic alopecia (AGA), which is mainly caused by an androgen action on the DP. Androgens alter the production of regulatory factors by the DP cells, causing anagen shortening and follicle miniaturization. Strong evidence has pointed out the human scalp HF sensitivity to androgens as the main reason for hair miniaturization, as opposed to androgens’ effect on hair from other body regions (eg, beard).^26^ Also, human scalp sensitivity to androgens is distinctive in comparison to other animals, which lack androgens-driven phenotypes (eg, prostate disorders or alopecia).^26^ In fact, mice do not suffer from AGA and key mechanisms controlling androgen-dependent HF miniaturization in the human scalp are not recapitulated in mice.^10,18^ Human and murine HF respond differently to distinct androgen and hormone stimulation. To circumvent this issue, an androgen-dependent mouse model expressing a human androgen receptor (AR) transgene, *K5-hAR*, was generated. Still, and noteworthy, in this model the human AR is expressed in basal epidermis and outer root sheath (ORS) mouse cells, and not in the DP,^27,28^ perhaps explaining why these mice do not exhibit a phenotype resembling human AGA.^29^ Therefore, caution is needed when resorting to the *K5-hAR* mice to validate drug therapies. In human AGA, two main factors determine terminal hair miniaturization to vellus: anagen growth shortening and decreased size of the DP and hair matrix.^30,31^ Studies using human DP spheroid cultures suggested that spheroid size is essential for HF inductivity, although not directly translating into thicker regenerated hair.^32^ In mice, selective ablation of DP cells in vivo showed that DP cell number determines the thickness and type of hair produced.^33,34^ The follicular papilla cell number and total papilla size are maximal by anagen VI, then decreasing by fibroblast migration out of the late anagen/early catagen papilla into the proximal connective tissue sheath.^34^ Thus, hair cycle-associated plasticity of the HF mesenchyme is likely clinically relevant.

Furthermore, not only hair cycle regulation differs between humans and mice, but also the stem cell niches (organization, markers, features) within the HF. Different HF stem and progenitor cells have been intensively investigated for their ability to generate tissue engineering applications to treat hair loss. However, the identification of specific biomarkers is still missing for their effective isolation and expansion. Even though mouse studies have significantly elucidated stem cell activation mechanisms during the hair cycle, major constraints still remain when translating these findings into tissue engineering strategies. Whereas the mouse anagenic bulge compartment (a reservoir of multipotent stem cells in the adult HF ORS) can be easily identified, within the human ORS there is a keratinocyte pool without apparent distinctive morphologies.^35^ Moreover, different biomarkers were found in mouse and human HF. For instance, CD34 and K15 markers, identified as murine bulge stem cell-specific markers and used for HFSC isolation,^36,37^ are not expressed in the human bulge region (CD34 is alternatively detected in the ORS).^38^ On the other hand, CD200, follistatin, and DIO2 appear to be human-specific markers of the bulge stem cells.^39,40,41^ Regarding the mesenchymal DP cells, integrin alpha 9^42^ and CD133^43^ have established cell surface markers for the mouse but not human DP. Importantly, the development of stem cell-based therapies for hair loss has been limited by the lack of robust human DP cell surface markers, which precludes their isolation by cell sorting.^44^ Therefore, another mesenchymal multipotent pool in the skin, the skin-derived progenitors (SKPs), has gained alternative attention in the hair research field. Mouse SKPs express neural crest biomarkers (eg, Slug, Snail, Twist, Pax3, Sox9, p75) and their role in DP renewal during the hair cycling has been demonstrated,^45-47^ as well as their dermal stem cell properties explored in bioengineering approaches.^45^ However, the study of human SKPs (hSKPs) has remained technically challenging and barely reported. The nestin, fibronectin, and vimentin markers expressed by hSKPs are also present in other skin mesenchymal populations,^48,49^ thus constraining the isolation, expansion, and validation of hSKPs’ therapeutical potential.

Finally, despite their similar structural layering and dramatic changes in dimension during the hair growth cycle, human and mouse skins differ in overall thickness. In mice, the epidermis is loose comprising only three cell layers (<25 µm), whereas the human epidermis is firm comprising many layers (>50 µm). Also, the human dermis is substantially thicker than the mouse dermis and contains fewer HFs.^50^ Therefore, their percutaneous absorption capacity is likely distinct, which should be considered in translational drug studies. Furthermore, immunological specificities during wound healing might dictate paradoxical outcomes in mice and humans. For example, mouse epidermal dendritic T cells secrete FGF-9 during wound healing, further triggering FGF-9 secretion by dermal fibroblasts.^51^ This mechanism explains the presence of HFs in mice but not in human scars.^52^ Such immunological specificities of the mouse skin are also disadvantageous to study alopecia areata (AA), an autoimmune disease that is the second most common cause of hair loss.^53^ Still, mouse models have provided invaluable means for studying the factors underlying immune regulation of this autoimmune skin disease.^54^ For example, the therapeutic effect of JAK inhibitors (Ruxolitinib and Tofacitinib) on AA was validated in the *C3H/HeJ* mouse model.^55^ However, *C3H/HeJ* mice develop spontaneous AA at a very low frequency, with histologic features that do not resemble those in human AA.^56^ Human AA is characterized by intra- and pre-follicular infiltration of CD4^+^ and CD8^+^ T lymphocytes. In murine AA, inflammatory cells infiltrate to the distal follicle and can reach the bulge. Key differences between the *C3H/HeJ* model and human AA pathobiology, genetics, and immunobiology have been reviewed,^56,57^ questioning the suitability of the mouse model to validate the efficacy of therapeutic compounds for human AA. For this reason, a humanized mouse model has been generated for preclinical studies.^58^

## Validation of Hair-inducing Capacity in Mouse Models

Animal models have been extensively used to functionally validate the ability of stem cell populations expanded in vitro to induce HF formation. Specifically, most hair inductive studies monitor the formation of “hair-like” structures upon implementation of bioengineered instructive mini-germs in mouse skin. HF bioengineering approaches have resorted primarily to human HFSCs or DPCs co-transplanted with mouse neonatal cells. Yet, successful generation of functional mature HFs exclusively from human adult HFSCs and dermal papilla cells (DPCs) has not been achieved (reviewed in Castro and Logarinho,^1^ Mohammadi et al,^59^ Nilforoushzadeh et al^60^). To cope with any constraining differences between mice and humans, human scalp skin xenografts in severe combined immunodeficiency (SCID) mice have been used. Noteworthy, this is currently the single preclinical assay that allows the establishment of a human hair cycle in vivo.^18^ Nude and SCID mutant mice have been routinely used in hair bioengineering approaches because: (i) nude mice constitute a unique model to study de novo hair formation, as they lack visible hair fibers, making xenografts easy to follow and study; (ii) SCID mice are deficient in T and B cells, which prevents the rejection of human scalp skin or human bioengineered transplants.^6,61^

Apart from xenografts, animal models have been also used to explore the efficacy of topical treatments to induce hair growth in vivo.^16^ Alternatively, human HF organ cultures (HFOCs) ex vivo have become increasingly popular in preclinical studies for hair growth/anti-AGA drugs.^62^ One common pitfall of preclinical drug validation in both mice and HFOCs is the fact that only gain/loss of anagen HFs can be monitored, and not the reversion of HF catagen and/or miniaturization.^62,63^ In fact, only maximally growing anagen VI HFs are used in HFOCs, which are thus unsuitable to study hair shaft elongation. Moreover, only occipital (not frontotemporal or vertex) HFs are used in HFOCs, which are more resistant to DHT-induced miniaturization (reviewed in Magerl et al^64^).

In sum, although several morphogenic and regenerative signaling pathways are evolutionarily conserved, species-specific differences may dictate if one given therapy will succeed in the human background. Studies of hair loss mouse mutants have been useful to (i) identify crucial genes on HF function, (ii) uncover the molecular mechanisms underlying hair morphogenesis, cycle, and pigmentation, and (iii) investigate the functional role of genes of interest through mutant phenotypes. However, mouse models might be also hindering the discovery of next-generation treatments for human hair loss as (i) they do not mimic the causal mechanisms behind human AGA and AA, and (ii) there are species-specific differences in HF growth and regulation as highlighted above.

## Emergent Human Cellular Models

**Figure 2.**
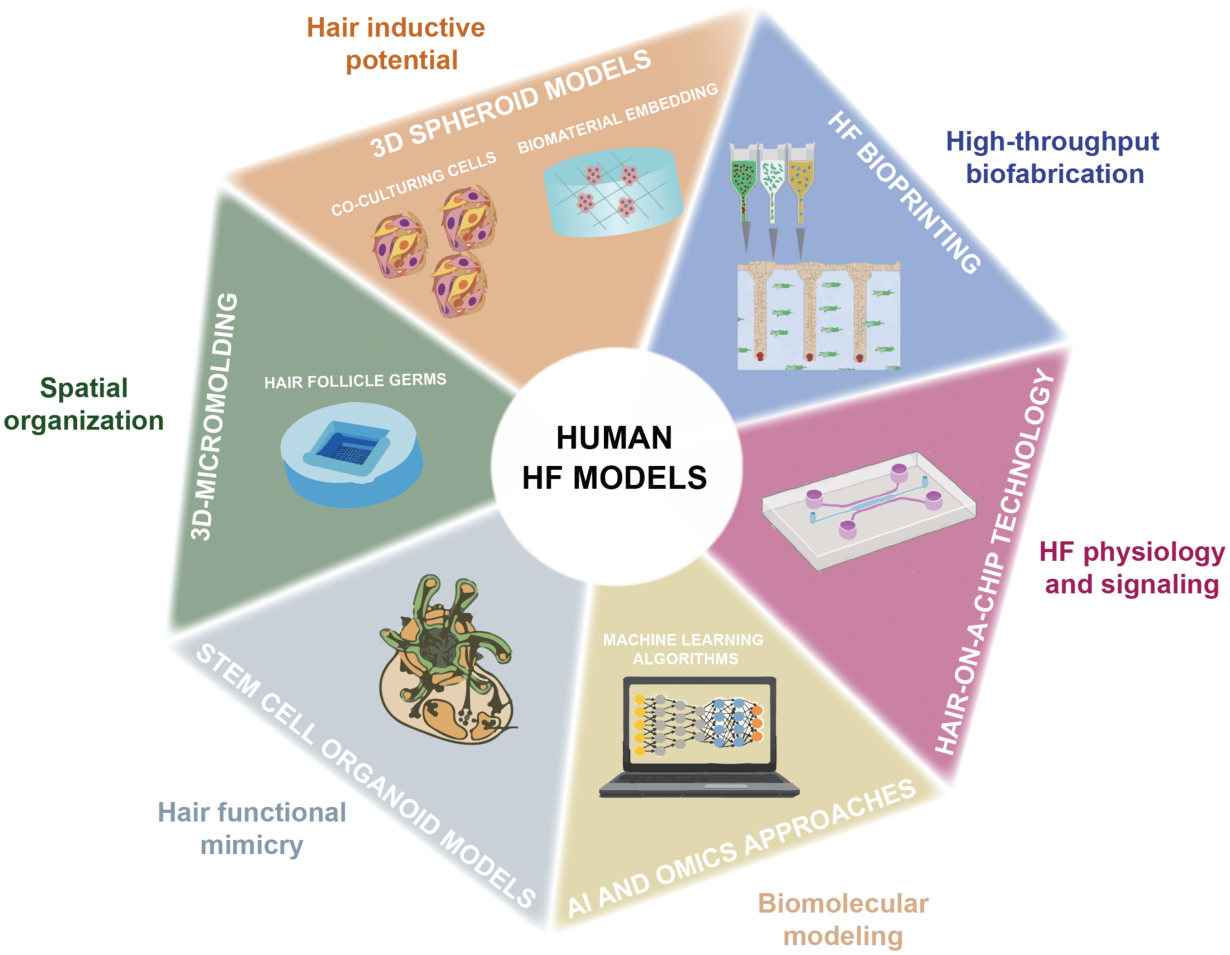
Advanced human cell-based models for hair research. Human cell-based models are needed to mimic HF’s structure and functional behavior. Different human engineering equivalents recapitulate specific traits of the native hair in vivo. Advances in bioengineering toward more sophisticated and reliable human HF 3D equivalents, as well as AI and Omics approaches, can accelerate drug development and bench-to-bedside research. HF bioprinting scheme adapted from ref ^72^. Organoid model scheme adapted from ref ^75^. Abbreviations: AI, artificial intelligence; HF, hair follicle.

Recent studies have disclosed innovative in vitro human models, based on bioengineered 3D co-culturing systems and organoids. These models aim to deliver a cellular array akin to the human HF, allowing to mimic the physiological response to drugs in high-throughput screenings. Moreover, the human cellular models answer to the 3Rs (replace, reduce, refine) guidelines on animal experimentation while providing an advanced alternative to safety testing. Nevertheless, it is unclear which specific factors are indeed crucial to maintain HF inductivity and cycling in vitro. Therefore, different combinations of factors and cellular architectures have been developed and tested (Fig. 2).

3D culture systems have long been applied in hair loss studies. 3D spheroid cultures using DPCs were shown to partially restore the transcriptional signature and hair inductive potential of human DPCs.^65,66^ Although DPCs are a well-established pool of cells for evaluating hair growth, they do not resemble the physiology of the whole HF. Therefore, 3D culture systems have further evolved toward the development of 3D-like structures that better recapitulate HF cell organization and communication.^67,68^

A method for efficient production of folliculoid keratinocytes-DP microtissues on a poly(ethylene-co-vinyl alcohol) (EVAL) surface has been developed, which in comparison to hanging drop methodology, generated hybrid spheroids with compartmented core (DP)-shell (keratinocytes) structure and able to grow hairs in vivo.^68^ More recently, a 3D co-culture system of hDP cells and hORS cells in an ultra-low attachment 96-well plate was established in which the two cell types constituted a polar elongated structure, that gradually increased while maintaining functional integrity as determined by the upregulation of hair growth-associated genes upon treatment with hair growth-promoting molecules.^67^ Furthermore, the combination of 3D printing technology with biomaterials has driven the large-scale production of architecturally relevant hair germs.^69^ 3D bioprinted multilayer scaffolds based on a gelatin/alginate hydrogel and different cell structures have also been used to mimic the DP microenvironment in the human scalp and thus reproduce a more physiological condition.^70^ Likewise, hair-like structures featuring human keratinocytes and spheroid-shaped human DP cells can be obtained by coaxial vertical bioprinting and culturing.^71^

Moreover, a biomimetic developmental approach of HF bioengineering was disclosed in which human HFs were generated within human skin constructs printed to incorporate keratinocytes and DP spheroids overexpressing the Lef-1 inductive factor. In addition, vascularization of hair-bearing human skin constructs before engraftment was shown to allow for efficient human hair growth in immunodeficient mice.^72^

More complex bioengineered human HF organoid models have emerged in recent years where pigmentation and innervation have been additionally included to physiologically resemble the human hair even deeper.^73-76^ In a recent approach, skin organoids containing pigmented HFs were obtained by a stepwise modulation of the TGF-β, BMP, and FGF signaling pathways in human-induced pluripotent stem cells (iPSCs) to co-induce differentiation into cranial epithelial cells and neural crest cells.^75^

Finally, organ-on-a-chip microfluidic technology applies cultured cells under fluid flow to recapitulate the physiology and pathophysiology of an organ. Skin and hair on-a-chip can thus be used to create patient-specific preclinical models.^77,78^ An EU COST Action for skin engineering and modeling (#21108) has just been approved to drive the development of cell-based and computational skin models, including the development of artificial intelligence (AI) models, for dermatological research. This holds strong potential to increase clinical outcomes and decrease animal experimentation in hair research.

The end goal of human hair-like structures’ bioengineering is the development of a disruptive hair loss replacement therapy. However, the complexity of the human HF still waits for an aesthetically relevant solution that brings added value over existing treatments such as the Follicular Unit Excision (FUE) transplant. Attention should now be given to the application of human bioengineered models to accelerate drug discovery and testing, as they more accurately forecast the physiology and drug response of human patients.^79,80^ Noteworthy, AI algorithm has been accelerating computer-assisted scalp diagnosis and automated hair loss count.^81^ Automated machine learning models could be also applied to other areas of hair research, including drug development and screening. In foreseeable future, AI might evolve to assist in patient-tailored treatments for hair loss, as well as to reprogram non-regenerating HFs to healthy cycling ones in combination with future whole-genome synthesis technology.

## Concluding Remarks

Mouse models have certainly contributed to the understanding of HF biology and the preclinical validation (efficacy and side effects) of hair loss treatments. The brief hair cycle experienced by the mouse HF is extremely advantageous in hair research, as it enables a hair cycle analysis across several regenerative loops and even during the animal’s life span. However, the structural and functional differences between mice and human HFs have forfeited the robustness of mouse models to meet the human hair loss therapeutic needs. Future research should therefore unequivocally address and establish the distinctive features between mouse and human hair in order to better extrapolate the results and predict clinical translation. Mouse and human HF regenerative capacity differs significantly, and human cell-based bioengineering in animal models has not completely succeeded, questioning the adequacy of mouse models to attain human HF regenerative therapies. In fact, the FDA-approved drugs for hair loss treatment available so far, Finasteride and Minoxidil, were identified based on their side effects on human hair growth during clinical trials, and not from studies in mice. It is thus possible that many hair treatment solutions developed over the past years and/or still under development may fail due to the inadequacy of animal models.

Although the clinical use of bioengineered HFs to treat hair loss disorders remains elusive, human cell-based bioengineered models should nevertheless be largely exploited in drug screening and testing. Indeed, high-throughput bioengineering models have been reported as easy and reliable to validate the hair growth-promoting effect of library molecules.^67,74,82^ The inclusion of DP cells in those bioengineered models allows for the screening of androgen-blocking agents. Hence, future efforts should concentrate on the development of in vitro human biomimetic platforms to be substantially explored on a preclinical basis.

In the near future, we anticipate that the technological developments will allow testing strategies in human cell models as the gold standard in the hair research field. Omics approaches to characterize the molecular signatures of HF cell populations, in both bald and non-bald scalp regions, should be given priority in order to accelerate bioengineering strategies. This tactic shift will help to demystify the holy grail of human HF regeneration, as well as will prevent the recurrent drawbacks in the later stages of clinical testing or the overlooking of potentially effective treatments that fail in mouse model testing.

## Data Availability

No new data were generated or analyzed in support of this research.
